# Decoding Microbiome’s Role in Prostate Cancer Progression and Treatment Response

**DOI:** 10.3390/diseases13090294

**Published:** 2025-09-05

**Authors:** Minas Sakellakis, Panagiota Resta, Evangelia Papagianni, Kassandra A. Procter, Irene Belouka, Katerina Gioti, Fragkiski Anthouli-Anagnostopoulou, Dimitrios Chaniotis, Apostolos Beloukas

**Affiliations:** 1 Department of Medicine, Jacobi North Central Bronx Hospital, The Albert Einstein College of Medicine, Bronx, NY 10461, USA; 2 Department of Biomedical Sciences, School of Health Sciences, University of West Attica, Egaleo, 12243 Athens, Greece

**Keywords:** prostate cancer, microbiota, gut microbiome, next-generation sequencing, microbial biomarker

## Abstract

Prostate cancer (PCa) is the most common genitourinary malignancy in men, with a multifactorial etiology influenced by genetic, environmental, and microbial determinants. Although the prostate was traditionally considered sterile, advances in microbiome research have challenged this view, revealing potential links between microbial communities and PCa development, progression, and treatment response. This review synthesizes evidence on the gut, urinary, seminal fluid, and prostatic microbiomes, highlighting their potential contributions to PCa pathogenesis and therapeutic outcomes. Key studies utilizing next-generation sequencing (NGS), whole-genome sequencing (WGS), PCR, and metagenomic analyses have identified specific bacterial and fungal taxa associated with Pca; however, findings remain inconsistent across methodologies and cohorts. Microorganisms such as *Propionibacterium acnes* and *Pseudomonas* spp. may modulate inflammation, immune responses, and resistance to androgen-deprivation therapy. Further research is required to determine whether microbial signatures can serve as reliable biomarkers for early detection, prognosis, or novel therapeutic strategies in PCa management.

## 1. Introduction

Prostate cancer (PCa) represents the most frequently diagnosed malignancy of the male genitourinary tract and represents a major global public health burden. In 2020, approximately 1.41 million new cases were diagnosed, and PCa accounted for 375,304 deaths worldwide, corresponding to nearly 8.6 million disability-adjusted life years (DALYs) [1,2]. In the United States alone, 299,010 new PCa cases and 35,250 deaths were projected for 2024 [3]. Despite a relatively high five-year survival rate, PCa remains a significant contributor to global cancer-related mortality. The introduction of prostate-specific antigen (PSA) screening programs has facilitated the earlier detection of localized disease, but has also raised concerns about overtreatment, as many identified tumors may remain indolent and clinically insignificant if untreated [4]. Standard therapies, including radical prostatectomy and radiotherapy, are associated with substantial morbidity and impaired quality of life. Moreover, even with early-stage detection and treatment, many patients ultimately experience disease recurrence and progression to more aggressive phenotypes [5].

The prostate was historically considered a sterile organ. However, advances in sensitive microbiological techniques have challenged this paradigm, paralleling shifts in our understanding of microbial colonization in other sites once presumed sterile [6,7]. Increasing evidence now implicates microorganisms in the etiopathogenesis of diseases traditionally categorized as non-infectious, such as the associations of *Helicobacter pylori* with gastritis and peptic ulcer disease, *Tropheryma whipplei* with Whipple’s disease, and high-risk human papillomavirus (HPV) subtypes with cervical carcinoma [8,9,10]. The identification of both culturable and unculturable microorganisms in such environments suggests a potential role for microbes in the pathogenesis of other diseases historically regarded as non-infectious.

Emerging evidence supports the concept that microbe alterations can drive chronic inflammation (e.g., helicobacter pylori) by modulating local tissue immune microenvironments and triggering oncogenic cascades, such as metaplasia, dysplasia, and atypia, that facilitate tumorigenesis [10,11,12]. The impact of the tumor-associated microbiome on carcinogenesis, cancer progression, and responses to therapeutic interventions has become an area of significant research interest [13]. Furthermore, accumulating data suggest that the gut microbiome is not only linked to colorectal cancer tumorigenesis and prognosis but also across other malignancies as well [14,15]. Beyond statistical associations and prognostic implications, recent studies even suggest a potential causative role [16]. Importantly, they underscore the gut microbiome’s pivotal role in modulating host immune responses [17]. Some recent studies have also shown that the therapeutic manipulation of the gut microbiota via probiotic administration might be a promising strategy to enhance the efficacy of cancer immunotherapy [18]. Collectively, these findings support the hypothesis that both gut and tumor-localized microbial populations can substantially influence cancer development and progression.

Nevertheless, the contribution of microbiomes to PCa pathogenesis remains poorly defined. PCa is typically classified as an immunologically “cold” tumor, consistent with its limited responses to contemporary immunotherapeutic strategies [19]. While several mechanisms linking microbes to prostate carcinogenesis have been proposed, robust translational evidence is still lacking [20]. A key research priority is the identification of novel biomarkers that can reliably distinguish indolent from aggressive disease at diagnosis with high specificity and sensitivity, becoming the figurative holy grail of PCa research.

In this review, we systematically summarize current evidence on the associations between PCa and microbial communities within the gut (Section 2), urine, feces, seminal fluid, and prostatic secretions (Section 3), as well as prostate tissue microenvironments (Section 4). We also evaluate contemporary methodologies employed for microbiome detection. Our objective is to critically assess whether specific microbial taxa or microbiota signatures may serve as reliable biomarkers for PCa diagnosis or prognosis. The early detection and targeted modulation of relevant microbial populations could open new avenues for prevention and therapy.

## 2. Gut Microbiome and Prostate Cancer: Emerging Evidence and Therapeutic Implications

Investigation of the gut microbiome in PCa has become an essential research focus, supported by growing evidence of its potential role in cancer pathogenesis. In a prospective case-control study utilizing whole-genome next-generation sequencing (WGS), Colombos et al. analyzed the gut microbiota composition of 12 patients with intermediate or high-risk PCa compared with 8 individuals with benign prostate conditions. PCa patients exhibited a higher abundance of *Bacteroides massiliensis*, whereas controls showed an enrichment of *Faecalibacterium prausnitzii* and *Eubacterium rectale* [21]. *Faecalibacterium prausnitzii* plays a key role in maintaining gut homeostasis as an acetate-consuming species that produces butyrate and anti-inflammatory metabolites, including salicylic acid derivatives [22]. Similarly, *Eubacterium rectale* is a butyrate producer with established anti-inflammatory properties within the gut microenvironment [23].

Supporting these observations, Liu et al. characterized gut microbiota by sequencing theV3 and V4 hypervariable regions of the bacterial 16S rRNA gene in 21 PCa patients at matched hormone-sensitive and castration-resistant disease stages undergoing androgen deprivation therapy. Distinct microbial alterations were identified in castration-resistant PCa, including the enrichment of *Phascolarctobacterium* and *Ruminococcus* [24]. Pernigoni et al. provided complementary insights, proposing that androgen-producing gut microbiota significantly contribute to the progression toward castration-resistant prostate cancer (CRPC). Recently Wang et al. found that the gut microbiome member *Clostridium scidens* contains an enzyme that catalyzes the conversion of androstenedione to epitestosterone. The authors showed that the latter impacts the proliferation of androgen-dependent prostate cancer cell lines in vitro, while the responsible enzyme was elevated in patients who are not responsive to abiraterone therapy [25]. In murine models, gut microbiota depletion via antibiotic administration delayed the onset of CRPC, whereas fecal microbiota transplantation (FMT) from castration-resistant subjects conferred resistance to castration therapies in recipient mice. Conversely, the administration of *Prevotella stercorea* or FMT derived from hormone-sensitive PCa patients effectively suppressed tumor growth, highlighting potential microbiome-targeted interventions [26].

## 3. Beyond the Gut: Exploring Microbial Signatures in Urine, Feces, Seminal Fluid, and Prostatic Secretions in Prostate Cancer

Microbial compositions exhibit marked inter-individual variability, influencing metabolic processes, local and systemic inflammatory responses, and immune regulation. These characteristics suggest potential utility as non-invasive biomarkers for early cancer detection and risk stratification. Alanee et al. analyzed paired fecal and urine samples from 30 patients undergoing prostate biopsy for elevated PSA. Using 16S rRNA sequencing (V3–V5 region), distinct urinary microbial profiles were detected in 71.4% of PCa patients, clustering separately from controls. This clustering was associated with Gleason score in urine but not fecal samples. Urine from PCa was enriched in *Veillonella*, *Streptococcus*, and *Bacteroides* spp., while *Faecalibacterium*, *Lactobacilli*, and *Acinetobacter* spp. were reduced. By contrast, patients without PCa exhibited higher abundances of *Clostridium* XVIII & IV, *Lachnospira*, *Acetanaerobacterium*, and *Faecalibacterium* in urine samples. Fecal samples from PCa patients showed an increased abundance of *Bacteroides* species, although overall bacterial diversity was comparable [27].

Yu et al. further profiled expressed prostatic secretions (EPS), urine, and seminal fluid from PCa (n = 13) and benign prostatic hyperplasia (BPH; n = 34) patients by sequencing the 16S rRNA V3 region. PCa patients exhibited an enrichment of *Bacteroidetes*, *Alphaproteobacteria*, *Firmicutes*, *Propionicimonas*, *Lachnospiraceae*, and *Ochrobactrum*, whereas BPH samples were enriched in *Eubacterium* and *Defluviicoccus*. *Alphaproteobacteria*, often implicated in urinary tract infections, may contribute to inflammation in PCa. *Firmicutes*, associated with obesity and caloric metabolism, and *Propionibacterineae*, frequently linked to prostatitis, were also prominent. *Ochrobactrum*, an opportunistic pathogen, may indicate immune dysfunction. While *Lachnospiraceae* are generally beneficial butyrate producers, they have paradoxical associations with metabolic syndrome and diabetes. PCa patients showed reduced urinary *Escherichia coli* but an increased abundance of EPS and seminal fluid, along with elevated *Enterococcus* spp. in seminal fluid [28,29].

Shrestha et al. analyzed 129 urine samples (61 PCa, 63 benign biopsies, and 5 initially benign progressing to PCa) using a two-step PCR targeting the 16S rRNA V6 region. Cancer samples harbored slightly higher microbial diversity (67 vs. 60 genera), dominated frequently by *Corynebacterium*, *Staphylococcus*, or *Streptococcus*, with *Anaerococcus*, *Lactobacillus*, and *Actinobaculum* being prominent in selected samples. A cancer-associated cluster included urogenital pathogens such as *Streptococcus anginosus*, *Anaerococcus obesiensis*, *Anaerococcus lactolyticus*, *Varibaculum cambriense*, *Actinobaculum schaalii*, and *Propionimicrobium lymphophilum*, though *Propionibacterium acnes* (*P. acnes*) did not significantly differ between groups [30].

Hurst et al. employed NGS and qPCR in 215 patients, correlating urinary microbiota with D’Amico risk scores, clinical stage, PSA, and Gleason score. Total operational taxonomic unit (OTU) counts did not correlate with PCa risk groups. The bacterial characterization of urinary sediments revealed four novel bacteria with unassigned OTU sequences in the NCBI dataset, which were frequently found in the patient’s urine. These included *Porphyromonas*, *Varibaculum*, *Peptoniphilus,* and *Fenollaria* spp. All were detected in prostatic secretions, while *Varibaculum* and *Peptoniphilus* were detected in prostate tissue by qPCR. Anaerobic culture yielded 39 bacterial isolates from urine, as well as 8 isolates from PCa secretions. These mostly included *Firmicutes*, *Fenollaria*, and *Anaerococcus* species. Five anaerobic genera, including three of the novel isolates, were associated with PCa risk group in cancer tissue, urine sediment, and urine extracellular vesicles. Prostate secretions yielded microbes from *Porphyromonas*, *Staphylococcus*, *Streptococcus,* and *Cutibacterium* species [31]. Other studies confirmed distinct urinary microbiota in PCa compared with controls, even in the absence of tissue-level differences [32].

Liss et al. profiled rectal swabs from 105 patients (64 PCa, 41 non-PCa) using 16S rRNA V1–V2 sequencing. Distinct microbial signatures were found between cancer and non-cancer groups despite similar diversity. The enrichment of *Bacteroides* and *Streptococcus* in PCa patients suggested metabolic reprogramming favoring carbohydrate metabolism and reduced B-vitamin synthesis [33].

A comprehensive systematic review of 16 studies (n = 1486, including 9 PCa-focused studies) reported considerable heterogeneity in urinary microbiota across prostatic diseases. Despite this variability, certain bacterial taxa were consistently associated with PCa. For instance, increased abundances of specific phyla, genera, and species were observed in PCa patients compared to controls. Moreover, some bacterial species were linked to higher-grade disease, suggesting a potential role in PCa progression [34]. Wang et al. also showed that bacterial strains from the urine produced androgens and were able to promote prostate cancer cell growth via cortisol and prednisone metabolism [25]. Collectively, these findings underscore the intricate relationship between urinary microbiota and prostatic diseases, highlighting the need for further research to elucidate the potential of microbiome-related biomarkers in PCa diagnosis and prognosis.

## 4. Intratumoral and Intraprostatic Microbiome: Emerging Roles in Prostate Cancer Pathogenesis

Intratumoral microbial communities are increasingly recognized as key modulators of cancer initiation, progression, and therapeutic response. These microbes interact with host genomic stability, induce epigenetic alterations, and shape inflammation responses. Notably, intratumoral microbiota may exert dual effects: enhancing antitumor immunity or, conversely, promoting tumor progression through mechanisms such as reactive oxygen species (ROS) induction, T-cell exhaustion, and the creation of immunosuppressive microenvironments. Utilizing large-scale RNA-sequencing data integrated with clinical parameters, Ma et al. identified specific intratumoral bacteria correlating with immune pathways, PCa risk factors, and tumor aggressiveness. Bacteria such as *Pediococcus pentosaceus*, *Listeria monocytogenes*, *Lactobacillus crispatus ST1*, and *Bacillus halodurans* negatively correlated with Gleason scores, suggesting anti-tumor roles, whereas *Nevskia ramosa* positively correlated [35]. The former three have been associated with anti-tumor effects in various tumor models, while *Listeria monocytogenes* has been implicated in the activation of innate and adaptive immunity and with the release of pro-inflammatory cytokines [35,36]. *Bacillus halodurans* and *Nevskia ramosa* have not been previously associated with cancer. *Rhodococcus erythropolis PR4*, *Delftia acidovorans SPH-1*, *Methylobacterium radiotolerans JCM 2831*, *Stenotrophomonas maltophilia K279a*, and *Meiothermus silvanus DSM 9946* demonstrated a negative correlation with TNM staging, while no bacteria correlated positively. Interestingly, *Rhodococcus erythropolis PR4*, *Stenotrophomonas maltophilia K279a*, and *Meiothermus silvanus DSM 9946* are often observed in immunosuppressed patients with in-dwelling catheters. Overall, 234 microbes were associated with increased PSA levels, while the most strongly correlated bacteria were *Campylobacter concisus UNSWCD*, *Thermus thermophilus HB27*, and *Streptococcus pneumoniae SPN032672*. *Thermus thermophilus* produces L-asparaginase, which can pose anti-tumor effects in several cancer types. *Streptococcus pneumoniae* has been associated with an increased risk of lower esophageal adenocarcinoma risk, while *Campylobacter concisus* is pro-inflammatory in the esophagus. The most strongly negatively correlated microbes included *Xanthomonas albilineans GPE PC73*, *Herminiimonas arsenicoxydans*, and *Pseudarthrobacter chlorophenolicus A6*. *Gardnerella vaginalis 409-05*, *Nitrobacter hamburgensis X14*, and *Delftia acidovorans SPH-1* were the most significant microbes positively correlated with the numbers of dysregulated immune-associated genes. Moreover, these bacteria were associated with downregulated genes that control immune system activation, suggesting that they are likely to promote PCa by suppressing immune cell expression, rather than by promoting inflammation. PCa tissue microbe abundance was associated with regulatory T-cell expression. *Bradyrhizobium elkanii*, *Ochrobactrum anthropi ATCC 49188*, and *Bradyrhizobium japonicum* demonstrated the strongest negative correlation with androgen receptor expression, while *Escherichia coli ETEC H10407* and *Escherichia coli str. K-12 substr*. *MG1655* showed the strongest positive correlation. The latter bacteria are frequently associated with prostatitis. *Staphylococcus aureus* subspecies MW2, *Paraburkholderia phymatum STM815*, *Haemophilus parainfluenzae T3T1*, and *Pseudomonas putida F1* were the most strongly associated bacteria with stem cell gene expression in the PCa samples [35,36].

Feng et al. analyzed microbial content within PCa tissue from 22 men using host-derived whole-genome sequencing (WGS). Viral or other nonbacterial read counts were negligible. Dominant genera included *Escherichia*, *Propionibacterium*, and *Pseudomonas*. *Escherichia* spp. have been shown to promote PCa cell growth in vitro, while other studies have confirmed that *Propionibacterium acne* is more commonly found in the prostate tissue of patients with PCa compared to patients without PCa. No associations were found between microbial taxa and clinical presentation (low- vs. high-risk disease). Samples from African patients contained a higher abundance of bacteria, especially anaerobic, compared to Australian and Chinese cohorts. However, the authors noted that transrectal biopsy sampling in African patients cannot rule out the possibility of fecal contamination. While half of the core gut microbial genera were absent, *Acidovorax* and *Escherichia* species were significantly abundant. Total bacterial burden and *Eubacterium* species abundance were correlated with prostate tumor host hypermutation. The authors concluded that this correlation may potentially explain (at least partially) the aggressiveness of disease in African men [37].

Additional studies using genomic and 16S rDNA sequencing reported widespread microbial presence in 170 prostate tissue specimens from 30 patients undergoing radical prostatectomy. Validation with organism-specific PCR in 200 PCa patients confirmed microbial DNA in 87% of individuals, although only 37% of individual tissue cores were positive. On average, 4.5 microbial sequences were detected per patient (range 0–14). The most common sequences were identified as members of *Gammaproteobacteria*, *Alphaproteobacteria*, CFB group bacteria, Gram+ Low GC Content bacteria, *Betaproteobacteria*, and Gram+ High GC Content bacteria. The most frequently identified sequences in multiple patients were similar to *Acinetobacter*, *Escherichia*, *Pseudomonas*, *Methylophilus*, and *Streptococcus* spp. Overall, organism-specific PCR failed to detect several microorganisms previously considered to be common in the prostate, and no specific microbe sp. was associated with evidence of acute or chronic inflammation. These results suggest the presence of regional heterogeneity with respect to bacteria, and the absence of a ubiquitous “landmark” microbiome in the prostatic flora. The bacterial culture results from prostatic samples were not on par with the results from 16S rDNA PCR, suggesting that either 16S rDNA PCR samples were derived from non-viable bacteria or that most bacteria inside the prostate were unculturable [38]. Keay et al. analyzed 18 transperineal biopsy specimens from nine PCa patients using 16S rRNA PCR. Bacterial DNA was detected in 11/18 samples (8/9 patients). A single or dominant organism was identified in most cases, while some contained multiple taxa. Sequence comparisons with GenBank indicated that the predominant organisms were mainly *Escherichia* and *Bacteroides* [39].

Krieger et al. obtained prostate biopsies from 107 PCa patients and 170 patients with chronic prostatitis/pelvic pain syndrome, as well as numerous controls. Bacterial DNA sequences were detected in 19.6% of patients with PCa compared to 46.4% of those with chronic prostatitis [40]. Similarly, Hochreiter et al. analyzed 14 samples from 7 patients after radical prostatectomy and several samples from normal controls. The results ruled out the existence of a normal flora inside the prostate. They reported that the presence of bacteria and/or inflammation were localized and heterogeneous events in patients with PCa and other inflammatory conditions. The presence of inflammation was strongly associated with positive 16S rRNA-PCR results. Although methodological differences (e.g., primer selection) may partly explain discrepancies, these studies support the hypothesis that prostatic bacteria contribute to chronic inflammatory conditions and may play a role in carcinogenesis [41].

Alexeyev et al. assessed archival prostate samples from 325 patients with benign prostatic hyperplasia for bacterial 16S RNA by implementing bulk Sanger sequencing (implemented with BigDye™ Terminator Cycle Sequensing kit 1.1 (Applied biosystems, Foster City, CA, USA)) and evaluated whether it differed among patients who later developed PCa (n = 171) and those who did not (n = 181). Overall, they detected bacterial 16S RNA in 96/352 specimens. The most frequently identified microorganism was *Propionibacterium acnes*, which was detected in 23% of 16S RNA-positive patients. The presence of *P. acnes* was also associated with severe histological inflammation and the future development of PCa (OR 2.17, 95% CI 0.77–6.95). The second most common microorganism was *Escherichia coli*, which was found in 12 (12%) patients. Other isolates included *Pseudomonas* and *Actinomyces* species, *Streptococcus* mutans, as well as *Corynebacterium*, *Nocardioides*, *Rhodococcus*, and *Veillonella* species [42]. In another study, a microbiome profiling of tumor, peri-tumor, and non-tumor tissues from 16 radical prostatectomy specimens using ultra-deep pyrosequencing (V3–V5 region) revealed diverse bacterial communities. Several phyla, classes, and genera exceeded the 1% threshold, confirming a rich intraprostatic microbiota. *Propionibacterium* dominated overall, *Staphylococcus* was most abundant in tumor and peri-tumor tissues, and *Enterococcus* was almost exclusively found in non-tumor areas [43].

Davidsson et al. showed that *P. acnes* significantly increased PCa risk, supported by in vitro data demonstrating enhanced inflammation and cellular proliferation following bacterial co-culture [44]. In contrast, Yow et al. identified consistent microbial communities dominated by Enterobacteriaceae and Escherichia within high-grade tumors, though without evidence of active infection, highlighting a complex microbiota–host interplay [45].

The frequency in which *P. acnes* is isolated in prostate tissue samples from patients with PCa compared to patients without PCa was researched using both culture diagnostics and molecular techniques. A total of 100 cases and 50 controls were included in Davidsson’s S. et al. study [44]. *P. acnes* was cultured in roughly 60% of patients with PCa compared to 26% of the controls. Multivariate analysis revealed that the presence of *P. acnes* was associated with a four-fold increase in the odds of a diagnosis of PCa after adjusting for confounding factors such as age, smoking status, or calendar year of surgery. The researchers also conducted in vitro experiments in which they co-cultured *P. acnes* isolates with the PNT1A PCa cell line, and they reported increased cytokine/chemokine secretion and increased proliferation in infected cells [44]. Also, Chen et al. [46] used three RNA-seq sets in the Illumina (NGS) system to identify *P. acnes* in cancer samples. They underlined the fact that it did not detect any human–bacteria or human–virus fusion in any data set that may suggest that *P. acnes* species do not pose severe risks for the development of prostate cancer.

Yow et al. also applied 16S rRNA sequencing (V2–V3 and V4) to 20 high-grade tumor cores. The researchers identified a plethora of operational taxonomic units (OTUs) in every sample (mean number: 231.55, range: from 151 to 314) using 16S rRNA V4 hypervariable region. The family of *Enterobacteriaceae* was the most abundant taxa (70.1%), followed by the genus *Escherichia* (6.9%). A small proportion of the overall membership of the prostatic microbial community (18 OTUs) was present in 95% of samples and contributed to 84.6% of the relative abundance of the total communities. *Enterobacteriacae* and *Escherichia* abundance was consistent across samples. Analysis with 16S rRNA V2–V3 hypervariable region identified 117.95 OTUs (range: from 71 to 160) per sample. *Enterobacteriaceae* and *Escherichia* sequences were represented in every sample. *Enterobacteriaceae*, *Streptococcaceae*, *Staphylococcus*, *Moraxella*, *Escherichia*, *P. acnes*, and *Streptococcus pseudopneumoniae* were represented in 95% of the samples and constituted the core community within the samples. Total RNA sequencing detected human endogenous retroviral sequences, but there were no viral or bacterial transcripts; hence, there was no evidence for active infection [45].

A combined metagenomic–metatranscriptomic study of tumor and matched benign tissues from 65 patients identified *Escherichia*, *Propionibacterium*, *Acinetobacter*, and *Pseudomonas* as the core prostate microbiome. Microbial diversity did not differ between tumors and benign samples or across Gleason categories. Few viral sequences were detected, but *Pseudomonas* species were inversely associated with metastasis [47].

Alluri et al. [48] investigated periodontal pathogens in 90 prostate tissue samples from 30 men. Using real-time PCR, they concluded that *Fusobacterium nucleatum* was the only pathogen that showed a significant difference in the prostates that harbored cancer, chronic inflammation, and benign prostatic hyperplasia. A total of 50 prostate adenocarcinoma samples were also used in Banerjee’s et al. [49] research to define the microbiome (viral, bacterial, fungal, and parasitic) signatures associated with prostate cancer. Using PathoChip technology, a technology which combines both PCR (*Poxviridae*, *Reoviridae*, *Papillomaviridae*, *Herpesviridaeand*) and next-generation sequencing (NGS), they found a large number of bacteria (*Rickettsia*, *Mycobacterium*, *Bordetella*, *Mycoplasma*, *Sphingomonas* etc.), viruses, fungi (*Alternaria*, *Malassezia*, *Candida*, *Cladosporium*, *Trichosporon*, *Cladophialophora* etc.) and parasites (*Plasmodium*, *Trichinella*, *Sarcocystis*, *Babesia*, *Entamoeba*) that could be identified as diverse microbiome signatures associated with prostate cancer.

Finally, Miyake et al. screened 45 PCa tissue samples for sexually transmitted pathogens, detecting *Mycoplasma genitalium* predominantly in younger patients, though without correlation to inflammation severity [50]. Salachan et al. used whole-transcriptome sequencing in 106 PCa tissue samples, reporting associations of *Shewanella*, *Vibrio parahaemolyticus*, and *Microbacterium* spp. with tumor progression [51]. Similarly, Sarkar et al. compared PCa and benign prostatic hyperplasia (BPH) samples via 16S rRNA sequencing, finding *Cupriavidus taiwanensis* and *Methylobacterium organophilum* enriched in PCa, whereas *Kocuria palustris* and *Cellvibrio mixtus* were more abundant in BPH [52].

**Figure 1:**
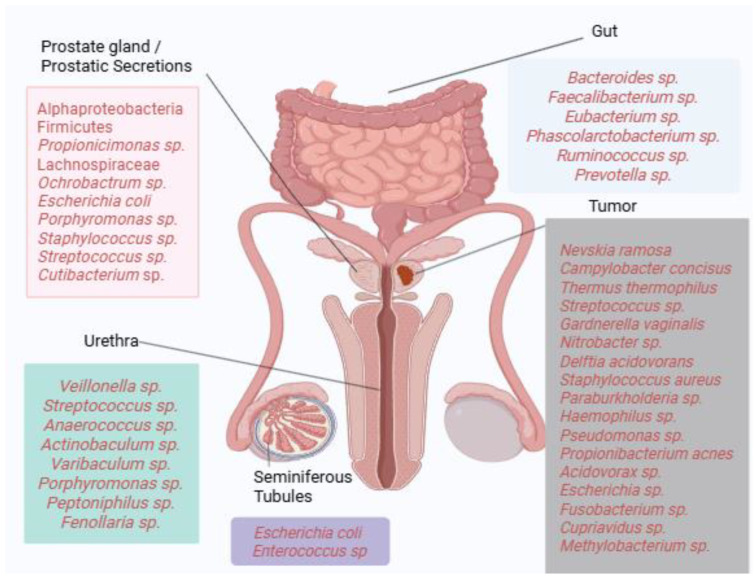
Microbial landscape of prostate cancer. Dominant bacterial taxa identified across gut, urinary, seminal, and prostatic tissue compartments are depicted. Where available, associations with clinical parameters such as Gleason score, TNM staging, PSA levels, or castration resistance are indicated in the main text. The figure highlights taxa recurrently reported across multiple studies while acknowledging the heterogeneity of findings (Created in https://BioRender.com).

Collectively, these findings illustrate the substantial heterogeneity of microbial communities detected across prostate tissues, with recurrent enrichment of taxa such as *Propionibacterium acnes*, *Escherichia*, and *Pseudomonas* in multiple studies. However, results vary considerably across methodologies, patient cohorts, and disease stages, highlighting the absence of a universally defined “core” prostatic microbiome. Figure 1 provides a visual summary of dominant bacterial taxa identified across different anatomical compartments, including gut, urine, seminal fluid, prostatic secretions, and tumor tissue. Where available, reported associations with clinical parameters such as Gleason score, PSA levels, TNM staging, or castration resistance are indicated in the main text. This schematic underscores both the diversity and complexity of microbial signatures linked to prostate carcinogenesis.

## 5. Mycobiome’s Role: Emerging Insights into Prostate Cancer

Emerging evidence indicates that fungal communities may also contribute to tumor biology. Certain fungal taxa are enriched in malignant tissues, suggesting potential roles in shaping the tumor microenvironment. The interaction between the mycobiome, bacterial microbiota, and host physiology opens novel avenues for cancer diagnostics and therapeutics [53]. In PCa, Wang et al. conducted a cross-sectional study of circulating plasma fungal microbiomes in PCa patients (n = 31) compared with age- and race-matched healthy controls (n = 34). Using the MR DNA (Shallowater, TX, USA) platform, the study revealed a pronounced enrichment of the fungal families *Filobasidiales* and *Pyronemataceae*, alongside the species *Cryptococcus ater*, exclusively in PCa patients. By contrast, diverse fungal classes and species were prominently elevated within the plasma microbiomes of the control group. Notably, a higher abundance of the genus *Bipolaris* was associated with lower PSA levels, while the increased representation of the class *Sordariomycetes* correlated with advanced pathological stages. These findings suggest that fungal signatures may hold diagnostic and prognostic value in Pca [53].

## 6. Virome

The potential role of the virome in prostate cancer has also been extensively investigated, although the overall conclusions so far remain inconclusive [54]. Several viruses have been detected in prostate tissue, including human papillomavirus (HPV), Epstein–Barr virus (EBV), herpes simplex virus type 2 (HSV-2), human herpesvirus 8 (HHV-8), cytomegalovirus (CMV), BK polyomavirus (BKV), and xenotropic murine leukemia virus-related virus (XMRV) [55,56,57,58,59,60,61]. Limitations related to detection methods, sample sizes, or study design variability lead to uncertainty regarding the consistency of these associations or the true frequency of infection. The HPV virus is of particular interest not only due to its proven tumorigenic role in other malignancies (cervix, vulva, anus, vagina, uterus, pelvis, head and neck) and its proximity to other urinary and anogenital sites, but also following preclinical evidence showing that viral proteins such as HPV E6/E7 have oncogenic potential by interfering with tumor suppressors like p53 and pRb [62,63]. Some studies have shown a statistical association of prostate cancer with sexually transmitted diseases, although a recent meta-analysis did not support this association [64]. High-risk HPV subtypes have been associated with prostate cancer in studies from Asian and some European populations (e.g., Greece, UK), but global results remain heterogeneous and non-confirmatory [11,54,55,56]. Moreover, XMRV has been isolated from prostate cancer tissue samples, but some evidence showed that the virus was formed in the laboratory and does not circulate in humans [65]. Although many viruses can interact with host proteins and result in genetic changes or immunological and inflammatory events that can favor the development or progression of tumors, it is believed that host genetic variations likely also play a role. For example, RNASEL R462Q polymorphism (that can lead to defective immunity and viral persistence) has been studied as a potential modulator of virome impact on prostate tissue [66,67]. Hence, the interplay between viral and host factors might exert pro-tumorigenic activity in prostate cancer, although more research needs to confirm this.

## 7. Discussion

Advances in sequencing technologies have fundamentally reshaped our understanding of microbial colonization, revealing diverse microbiome communities even in anatomical sites once considered sterile. These discoveries have spurred renewed interest in the role of gut, urinary, and tumor-associated microbiomes in PCa [7,10]. Although the studies reviewed here exhibit marked methodological and biological heterogeneity, along with some conflicting results, several consistent observations can be highlighted.

First, the increased abundance of *Bacteroides* spp. in the gut microbiome has been repeatedly associated with malignancy, while benign conditions were more often linked to beneficial genera such as *Faecalibacterium* and *Eubacterium* [21,22]. Although gut microbial composition has not generally correlated with Gleason score, some taxa have been associated with resistance to androgen-deprivation therapy. These findings, however, are limited by small sample sizes and should be interpreted cautiously [24].

Urinary microbiota have also been implicated in prostate carcinogenesis. Commonly reported genera include *Bacteroides*, *Firmicutes*, *Varibaculum*, and *Streptococcus*. The role of *Propionibacterium acnes* remains controversial, with inconsistent associations across different cohorts [28,30,34]. The frequent detection of identical bacterial species in both prostatic secretions and seminal fluid as well as urine samples suggests potential biological interplay among these compartments [28,29]. Despite variability between studies, *Escherichia*, *Propionibacterium*, *Pseudomonas*, and *Acinetobacter* are among the taxa most frequently identified in prostate tissue and peri-tumoral regions [35,36]. While *Enterobacteriaceae* are also commonly reported, some evidence suggests they may form part of the normal prostate microbiota and could exert protective or even antitumor effects [45].

The inconsistencies across studies highlight the complexity of microbial involvement in PCa and suggest that no single microorganism is solely responsible for tumor initiation or progression [68]. A “hit-and-run” model, in which pathogens contribute to early carcinogenesis but are no longer detectable in advanced stages, remains a plausible hypothesis [68,69,70].

Importantly, methodological heterogeneity likely explains many of the inconsistencies observed across studies. Differences in sequencing platforms, amplified regions, and bioinformatic pipelines can profoundly affect microbiome profiling and taxonomic resolution. As summarized in Table 1, most studies relied on amplicon-based 16S rRNA sequencing (e.g., Illumina MiSeq/HiSeq platforms targeting V3–V4, V3–V5, or V6 regions), which provides genus- or species-level resolution but limited functional insight. In contrast, a smaller number of studies employed whole-genome or whole-transcriptome sequencing, enabling a higher resolution of microbial taxa and functional pathways, albeit at greater cost and complexity. Table 2 further illustrates the variability in PCR-based methods, with diverse primer sets targeting different microbial taxa, including bacteria, viruses, and fungi. Such differences not only influence the detection of specific organisms (e.g., *Propionibacterium acnes*, *Escherichia coli*, or viral sequences) but also limit comparability across studies.

Taken together, these observations highlight the urgent need for standardized methodologies in prostate cancer microbiome research. The harmonization of sequencing approaches, target regions, and analytical pipelines will be essential to reduce variability and enable reproducibility. Only through methodological standardization and integration of multi-omic platforms (16S, shotgun metagenomics, metatranscriptomics, and targeted PCR) can reliable microbial biomarkers be identified and validated for clinical application in prostate cancer.

## 8. Conclusions

Microbiome research has considerably expanded our understanding of the potential role of microbiomes in prostate cancer (PCa) pathogenesis, progression, and treatment response. Across gut, urinary, seminal, and prostatic compartments, recurrent associations have been identified with taxa such as *Bacteroides*, *Escherichia*, and *Propionibacterium acnes*. However, significant heterogeneity persists, reflecting differences in patient cohorts, sampling methods, and analytical platforms. Importantly, no single microorganism has been established as a universal hallmark of PCa, and causality remains unproven.

Nevertheless, accumulating evidence suggests that microbial signatures may influence carcinogenesis through chronic inflammation, the modulation of local immune responses, and metabolic interactions, with implications for resistance to androgen-deprivation therapy and responsiveness to immunotherapy. This highlights the potential utility of microbiome profiles as biomarkers for diagnosis, risk stratification, and therapeutic guidance.

To translate these insights into clinical practice, future research should prioritize methodological standardization across sequencing platforms, genomic regions, and bioinformatic pipelines. Integration of multi-omic approaches—including 16S rRNA sequencing, shotgun metagenomics, metatranscriptomics, and targeted qPCR—will be essential to improve resolution, reproducibility, and functional interpretation. Large, prospective, and ethnically diverse cohorts will be required to validate candidate microbial biomarkers and clarify their mechanistic roles in PCa.

Ultimately, a standardized, systems-level approach to the prostate cancer microbiome could yield robust biomarkers for early detection and prognosis while paving the way for microbiome-targeted interventions as adjuncts to existing therapies. Such strategies hold promise for improving patient stratification, optimizing treatment response, and advancing the personalized management of prostate cancer.

## Figures and Tables

**Table 1:** Overview of next-generation sequencing (NGS) platforms used in prostate cancer microbiome studies.

Reference	Anatomical Site/Region	Experimental Platform	Type of Library (Amplicon-Based vs. Whole-Genome)	Region Amplified	Taxonomy Level
Golombos DM. et al. [21]	Gut	Illumina NextSeq	Whole-genome		Species
Liu Y et al. [24]	Gut	Illumina MiSeq	Amplicon-based	16S rRNA (V3–V4 region)	Genus
Alanee et al. [27]	Urine and fecal	Illumina MiSeq	Amplicon-based	16S rRNA (V3–V5)	Species
Shrestha E. et al. [30]	Urine	Illumina HiSeq	Amplicon-based	16S rRNA (V6)	Species
Hurst R. et al. [31]	Urine	Illumina MiSeq	Whole-genome		Species
Feng Y. et al. [37]	PCa tissue	Illumina HiSeq	Whole-genome		Genus
O. Alexeyev et al. [42]	PCa tissue	Cycle sequencing (Applied Biosystems)	Amplicon-based	16S rRNA	Species
Cavarretta I. et al. [43]	PCa tissue	Pyrosequencing	Amplicon-based	16S rRNA (V3–V5)	Species
Yow MA et al. [45]	PCa tissue	Illumina MiSeq	Amplicon-based	16S rRNA (V2–V3 and V4)	Species
Feng Y. et al. [47]	PCa tissue	Illumina HiSeq	Whole-genome		Genus
Wang X. et al. [49,53]	Plasma	sequenced by MR DNA (Shallowater, TX, USA).	Amplicon-based	fungal ITS sequence	Species
Banerjee S, et al. [49]	PCa tissue	sequenced by TransPlex (Sigma-Aldrich, St. Louis, MO, USA)	Whole-genome and transcriptome amplification		Genus/species
Chen Y. et al. [46]	PCa tissue	Illumina mRNA-seq	Whole genome		Species
Gonçalves et al. [32]	Urine, glans, PCa tissue	Illumina MiSeq	Amplicon-based	16S rRNA (V3–V4)	Genus
Salachan et al. [51]	PCa tissue	Illumina NovaSeq or NextSeq 500.	Whole-transcriptome profiling		Species
Sarkar et al. [52]	Prostate (BPH & PCa)	Ion GeneStudio S5 System	Amplicon-based	16S rRNA	Species

**Table 2:** Overview of PCR-based methodologies applied in prostate cancer microbiome studies.

Reference	Target Region	PCR Type	Primers	Region Amplified	Taxonomy Level
Yu H et al. [28]	Prostatic secretions, urine, and seminal fluid	Conventional PCR and qPCR	41F: 5′-GTATTACCGCGGCTGCTGG-3′; 534R: 5′-ACTCCTACGGGAGGCAGCAG-3′ with a 40-bp GC clamp: 5′CGCCCGCCGCGCGCGGCGGGCGGGGCGGGGGCACGGGGGG3′	16S rRNA (V3)	Species
			VS8F: 5′-GGCGGATTAGACTTCGGCTA-3′, VS9R: 5′-CGTTTTGGCACTATTTGCCC-3′	*E. coli*	
			Ent1F: 5′-TACTGACAAACCATTCATGATG-3′, Ent2R: 5′-AACTTCGTCACCAACGCGAAC-3′	*Enterococcus*	
Shrestha E. et al. [30]	Urine	qPCR Real-time PCR	F: 5′-GCGTGAGTGACGGTAATGGGTA-3′ R: 5′-TTCCGACGCGATCAACCA-3′.	*P. acnes*	Species
			F: 5′-CATTGATAACGAAGCTCTTTACGAT-3′ R: 5′-GCATGTTGTGCCGGACATAACCAT-3′	*T. vaginalis*	
Hurst R. et al. [31]	Urine	qPCR	F: 5′-GCGAACAAACGTCAAGGAAC-3′ R: 5′-GCCTTTCCATTGAGGGCTTC-3′	*Fenollaria* sp.	Species
			F: 5′-CACCGAAGACCAAGGCGTTA-3′ R: 5′-GGTGCCGACCGTAGAAACTT-3′	*Peptoniphilus* sp.	
			F: 5′-GCGTTGATGAAGCCCTCTCTAT-3′ R: 5′-ACCTTTAGCCTTAGGACGGAA-3′	*Peptoniphilus harei*	
			F: 5′-CGCTCGCAAACAGGTTGAAT-3′ R: 5′-GGGCAGCATTTTCCGAAGC-3′	*Varibaculum* sp.	
			F: 5′-CGATCATACCTGGACGAGCC-3′ R: 5′-TCGGCTACATACGTGGTTGG-3′	*Porphyromonas asaccharolytica*	
			F: 5′-TCTGAATGGGCAGTTGAAGGA-3′ R: 5-AGCTTCCCCTCCTTCTTTCTT-3′	*Fusobacterium nucleatum*	
			F: 5′-ATGAGCCCGATGAAGGTTCG-3′ R: 5′-CTACCGCAGAGGCAACTACC-3′	*Propionimicrobium lymphophilum*	
			F: 5′-GGATGACCTTGGTGGGGTAG-3′ R: 5-CACACAAATGGTGGTCACGG-3′	*Cutibacterium acnes*	
Sfanos et al. [38]	PCa tissue	Conventional PCR	1E 5′-TCAAATGAATTGACGGGGGC-3′ 13B 5′-AGGCCCGGGAACGTATTCAC-3′	16S rDNA	Species
		Conventional PCR	CtMOMP-F: 5′-CCTGTGGGGAATCCTGCTGAA-3′ CtMOMP-R: 5′-GTCGAAAACAAAGTCACCATAGTA-3′	*C. trachomatis*	Species
			PA-F 5′-GGGTTGTAAACCGCTTTCGCTG-3′ PA-R 5′-GGCACACCCATCTCTGAGCAC-3′	*P. acnes*	
			BTUB9-F 5′-CATTGATAACGAAGCTCTTTACGAT-3′ BTUB2-R 5′-GCATGTTGTGCCGGACATAACCAT-3′	*T. vaginalis*	
		Nested PCR	BKV-F 5′-TTTTGGAACCTGGAGTAGCTCAGAGGTTT-3′ BKV-R 5′-GCTTGACTAAGAAACTGGTGTAGAT-3′ BKVnes-F (nes) 5′-CCTCTTTGCCCAGATACCCTGTACT-3′ BKVnes-R(nes) 5′-GAGAATCTGCTGTTGCTTCTTCATC-3′	BKV	
		Conventional PCR	EBV-EBER-F 5′-CCCTAGTGGTTTCGGACACA-3′ EBV-EBER-R 5′-ACTTGCAAATGCTCTAGGCG-3′	EBV	
			CMVpp65-375-F 5′-CATCAACGTGCACCACTACC-3′ CMVpp65-562-R 5′-ACACGAACGCTGACGTGTAG-3′	CMV	
			GP5+-F 5′-TTTGTTACTGTGGTAGATACTAC-3′ GP6+-R 5′-GAAAAATAAACTGTAAATCATATTC-3′	HPV	
		Nested PCR	GAG-O-F 5′-CGCGTCTGATTTGTTTTGTT-3′ GAG-O-R 5′-CCGCCTCTTCTTCATTGTTC-3′ GAG-I-F (nes) 5′-TCTCGAGATCATGGGACAGA-3′ GAG-I-R (nes) 5′-AGAGGGTAAGGGCAGGGTAA-3	XMRV	
Keay S. et al. [39]	PCa tissue	Conventional PCR and nested PCR	F: 5′-CACAAGCGGTGGAGCATGTGGTT-3′ R: 5′-CCTACGGYTACCTTGTTACCACT-3′, where Y equals C or T F: 5′-GGAATTCTGCAACGCGAAGAACCTTACCT-3′ R: 5′-GCGGATCCTGGTKTGACGGGCGGTGTGTA-3′, where K equals G or T	16s rRNA	Species
Hochreiter W. et al. [41]	PCa tissue	Conventional PCR and nested PCR	1492RPL: 5′-GGTTACCTTGTTACGACTT-3′ 8FPL: 5′-AGTTTGATCCTGGCTCAG-3′ 91E: 5′-TCAAAKGAATTGACGGGGGC-3′ 13B: 5′-AGGCCCGGGAACGTATTCAC-3′	16s rRNA	Genus
Alexeyev O. et al. [42]	PCa tissue	Conventional PCR and nested PCR	16SFa: 5′-GCTCAGATTGAACGCTGG-3′ 16SFb: 5′-GCTCAGGAYGAACGCTGG-3′ 16SR: 5′-TACTGCTGCCTCCCGTA-3′ 16SFac: 5′-CAGATTGAACGCTGG-3′ 16SFbc: 5′-CAGGAYGAACGCTGG-3′ 16SRc:5′-TGCTGCCTCCCGTA-3′	16s rRNA	Species
Cavarretta I. et al. [43]	PCa tissue	Conventional PCR, nested PCR, and qPCR	16S-F8: 5′-AGAGTTTGATCCTGGCTCAG-3′ 16S-R1093: 5′-GTTGCGCTCGTTGCGGGAC-3′ 16S-F331: 5′-ACT CCT ACG GGA GGC AGC-3′ 16S-R920: 5′-CCG TCA ATT CMT TTG AGT TT-3′ 926F: 5′-AAA CTC AAA KGA ATT GAC GG-3′ 1062R: 5′-CTC ACR RCA CGA GCT GAC-3′	V3-V5 16s rRNA	Species
Alluri LSC, et al. [48]	Prostate Gland	Real-time qPCR	Commercially available website-integrated DNA technology (IDT)	Not specified	Species
Banerjee S, et al. [49]	PCa tissue	Conventional PCR	F: 5′-TAGGTGCCAACCTATGGAACAGA-3′ R: 5′-GGAAAGTCTTTAGGGTCTTCTACC-3′	Polyomavirus FP	Genus /species
			F: 5′-TACCAGTGGAATGTTCTACCNCARGGN-3′ R: 5′-ATCAGATCCTACTAACDRTCRTCCATRTA-3′	Retrovirus FP	
			F: 5′-CCAGACGGCAAGGTTTTTATCC-3′ R: 5′-TTGAGCTCTAGGCACGTTA-3′	KSHV FP	
			F: 5′-AGT AGT GTT GCA GCA CTA TAT TGG-3′ R: 5′-ATG CCC ATT GTA CCA TTT CTG AC-3′	HPV18_E1 FP	
			F: 5′-ACC ATT ATC CCC ATA CAA CAA TG-3′ R: 5′-CTC TTG GTG ATA TGG AAA TGT TGG-3	*Helicobacter* cagA FP	
			F: 5′-GCG AAT CCT TTT AAA GCC GGT CTC-3′ R: 5′-TGT TAC CGA CTT TCA TGA CGT G-3′	*Mycobacterium* FP	
			F: 5′-TCG TTA CCT GTG TTA GCC AGA G-3′ R: 5′-TCC TTA GAC TCA TAC AGA TAT GCC-3′	*Schistosoma* FP	
			F: 5′-TGT GAC CAA AGC AGT CAT TCG-3′ R: 5′-GTG TGT ATG TGT GTG GAA TAA CC-3′	*Trypanosoma* FP	
			F: 5′-TTC AGA AGG AAG TAC CAG TAG G-3′ R: 5′-TGA TTG TGC AAA TCC GAA TCG AG-3′	*Trichosporon* FP	
			F: 5′-TGC GTT TGA ATA CTA CAG CAT GG-3′ R: 5′-CTT CGC AGT TGT TTG TCT CCA G-3′	*Plasmodium* FP	
			F: 5′-CAA GTG TCT GCC TTA TCA ACC TTC-3′ R: 5′-TGC CTT CCT TGG ATG TGG TAG-3′	*Trichinella* FP	
			F: 5′-GAT AGC CGT GTT AAT TCT ATG GC-3′ R: 5′-TCA GAA ACT TGA ATG ATC CAT CGC-3′	*Sarcocystis* FP	
Miyake et al. [50]	PCa tissue	Conventional PCR	F: 5′-GCGACGTCATCGGTAAATACC-3′ R: 5′-CGCCATACGGACGATGGT-3′	*Neisseria gonorrhoeae*	Genus
			F: 5′-TGATGTATCCAGCCCAAATGC-3′ R: 5′-AATCCAGTTCTTCTCTGCCTCTCTAC-3′	*Chlamydia trachomatis*	
			F: 5′-GAGAAATACCTTGATGGTCAGCAA-3′ R: 5′-GTTAATATCATATAAAGCTCTACCGTTGTTATC-3′	*Mycoplasma genitalium* (short amplicon)	
			F: 5′-AGTTGATGAAACCTTAACCCCTTGG-3′ R: 5′-CCGTTGAGGGGTTTTCCATTTTTGC-3′	*Mycoplasma* *genitalium* (long amplicon)	
			F: 5′-GATCACATTTCCACTTATTTGAAACA-3′ R: 5′-AAACGACGTCCATAAGCAACTTTA-3	*Mycoplasma hyorhinis*	
			F: 5′-ACACCATGGGAGCTGGTAAT-3′ R: 5′-CTTCTCGACTTTCAGA-3′	*Ureaplasma urealyticum*	
			F: 5′-CACAGTTATGCACAGAGCTGC-3′ R: 5′-CATATATTCATGCAATGTAGGTGTA-3′	HPV16	
			F: 5′-CACTTCACTGCAAGACATAGA-3′ R: 5′-GTTGTGAAATCGTCGTTTTTCA-3′	HPV18	

## Data Availability

Not applicable since no new data were created.

## Abbreviations

The following abbreviations are used in this manuscript:

BPH	Benign prostatic hyperplasia
CI	Confidence interval
CRPC	Castration-resistant prostate cancer
EPS	Expressed prostatic secretion
FMT	Fecal microbiota transplantation
NGS	Next-generation sequencing
OTU	Operational taxonomic unit
PCR	Polymerase chain reaction
PSA	Prostate-specific antigen
TNM	Tumor–node–metastasis classification
